# Identification of “regulation of RhoA activity panel” as a prognostic and predictive biomarker for gastric cancer

**DOI:** 10.18632/aging.202179

**Published:** 2020-12-03

**Authors:** Wenwen Huang, Songhui Zhao, Cheng Zhang, Zhongwu Li, Sai Ge, Baofeng Lian, Hui Feng, Kai Wang, Ruihua Xu, Jiafu Ji, Jing Gao, Weiwei Shi, Lin Shen

**Affiliations:** 1 Department of Gastrointestinal Oncology, Key Laboratory of Carcinogenesis and Translational Research (Ministry of Education, Beijing), Peking University Cancer Hospital and Institute, Beijing 100142, China; 2 OrigiMed Inc., Shanghai 201112, China; 3 Department of Pathology, Key Laboratory of Carcinogenesis and Translational Research (Ministry of Education/Beijing), Peking University Cancer Hospital and Institute, Beijing 100142, China; 4 Shanghai Junshi Biosciences Co., Ltd, Shanghai 201203, China; 5 State Key Laboratory of Oncology in South China, Department of Medical Oncology, Sun Yat-sen University Cancer Center, Collaborative Innovation Center for Cancer Medicine, Guangzhou 510060, China; 6 Department of Gastrointestinal Surgery, Key Laboratory of Carcinogenesis and Translational Research (Ministry of Education/Beijing), Peking University Cancer Hospital and Institute, Beijing 100142, China; 7 National Cancer Center/National Clinical Research Center for Cancer/Cancer Hospital and Shenzhen Hospital, Chinese Academy of Medical Sciences and Peking Union Medical College, Shenzhen 518116, China; 8 Department of Thoracic Surgery, Shanghai Pulmonary Hospital, School of Medicine, Tongji University, Shanghai 200433, China

**Keywords:** regulation of RhoA activity, gastric cancer, prognosis and predictive biomarker, tumor migration, tumor microenvironment

## Abstract

RhoA is a member of the RHO family GTPases and is associated with essential functions in gastric cancer. In this study, we identified a gastric cancer biomarker, termed the “regulation of RhoA activity panel” (RRAP). Patients with gastric cancer from The Cancer Genome Atlas database were divided into training (N=160) and validation (N=155) cohorts. A cohort of 109 Chinese gastric cancer patients was utilized as an independent validation. Patients with mutated RRAP showed significantly better overall survival than patients with wild type RRAP. We also analyzed the association between RRAP and the migration capacity, immune-related signatures, and the tumor microenvironment. RRAP-mutant tumors had a significantly lower degree of lymph node metastasis and lower activities of migration-related pathways. These tumors also showed significantly increased immune cell infiltration and cytotoxic activity. Furthermore, two independent patient cohorts who received immune checkpoint blockade therapy were assessed for RRAP mutant status. As expected, for both immunotherapy cohorts, higher response rates to immune checkpoint blockade therapy were observed in patients with RRAP-mutant tumors than in patients with wild type RRAP tumors. Overall, this study indicates that the RRAP gene set is a potential biomarker for gastric cancer prognosis and therapeutic selection.

## INTRODUCTION

Gastric cancer is one of the most frequently occurring and lethal malignancies [1]. The Lauren classification divides gastric cancer into 2 main subtypes, intestinal and diffuse [2]. However, this classification provides limited guidance for disease prognosis and treatment decisions. Global efforts to characterize gastric cancer at the molecular level from the perspective of cancer genomics and transcriptomics have been made, including those from The Cancer Genome Atlas (TCGA) [3] and the Asian Cancer Research Group [4]. Gastric cancer patients are classified into 4 subtypes based on gene expression profiling; each subtype exhibits distinct patterns of molecular alterations, disease progression, and prognosis [4]. Although these large-scale efforts have provided comprehensive insights into gastric cancer, they have not translated into a clinical benefit. A genomic-based molecular biomarker with prognostic and/or therapy predictive value is still needed for gastric cancer.

Among the various genomic alternations that occur in gastric cancer, *RHOA* mutation plays a critical role in the development and progression of cancer by regulating actin organization [5], cell migration [6], cytokinesis and the cell cycle [7]. Recent studies have also suggested its potential role in modulating the tumor microenvironment (TME) of cancers [8, 9]. Although the overexpression of RhoA has been frequently recognized in various cancers and was found to be significantly associated with poor prognosis in gastric cancer [10], similar overall survival (OS) rates were nevertheless observed between patients with *RHOA* mutant and wild-type gastric cancers [11, 12]; therefore, the prognostic value of this gene mutation is poor. In 2017, Shi et al. [13] established that mutations within the gene set designated as the “regulation of RhoA activity pathway” were associated with better progression-free survival (PFS) and overall survival in HER2+ breast cancer patients. This gene set includes RhoA, as well as guanine nucleotide exchange factors and GTPase-activating proteins [14], both of which are involved in regulating RhoA activity. Furthermore, altered RhoA signaling has been reported in gastric cancers, especially in the diffuse type gastric cancer [3, 15–21]. However, the clinical significance of this pathway in overall gastric cancer remains unresolved. Mutations in “regulation of RhoA activity pathway” gene set may be involved in the prognosis and therapeutic prediction of gastric cancer through their effect on RhoA and its effector molecule activity.

Based on the pathway changes of RhoA activity, we developed a statistically optimized gene subset as a biomarker by applying a genetic algorithm to a training gastric cancer cohort obtained from the gastric cancer dataset of TCGA. We validated this biomarker in a nonoverlapped TCGA-validation cohort and in an independent Chinese gastric cancer (CGC) cohort. The association of this biomarker with lymph node metastasis, migration-related pathways, immune-related signatures, and the TME was assessed to glean insights into possible related mechanisms. Inspired by its effect on the TME, we collected 2 independent cohorts of gastric cancer patients (denoted as IM1 and IM2) who received immune checkpoint blockade (ICB) treatment and revealed the potential predictive capability of this biomarker.

## RESULTS

### Prognostic biomarker “regulation of RhoA activity panel” for gastric cancer

**Figure 1:**
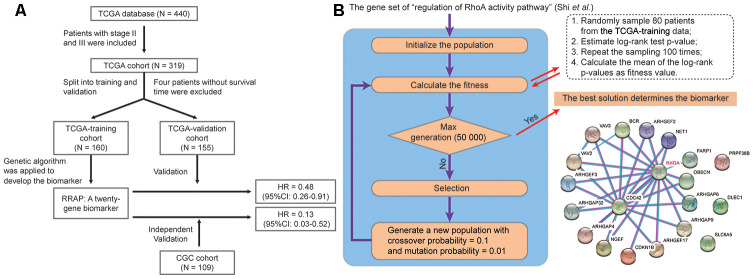
**Identification of RRAP biomarker.** (**A**) Outline of the cohort construction and analysis workflow. (**B**) RRAP selection. The right panel shows the interaction of genes among RRAP.

**Figure 2:**
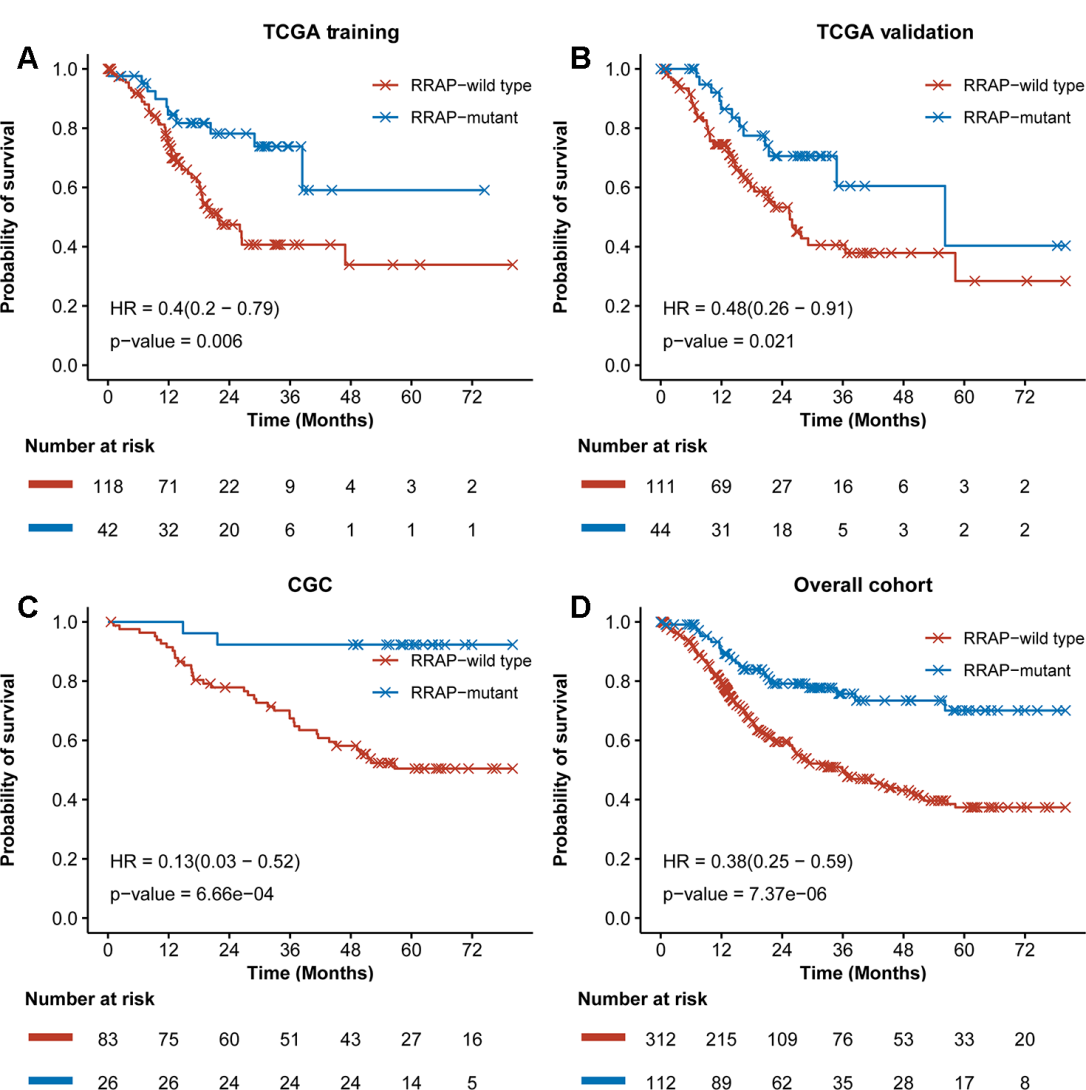
**Training and validation of RRAP.** Kaplan-Meier survival curves for RRAP-wild type and RRAP-mutant patients in the (**A**) TCGA training cohort, (**B**) TCGA validation cohort, (**C**) an independent CGC cohort, (**D**) and the overall cohort containing merged data from the three cohorts. P-values were estimated with the log-rank test, and hazard ratios (HRs) were estimated with the Cox model.

As shown in Figure 1, the potential biomarker “regulation of RhoA activity panel” (RRAP) was calculated from the TCGA training cohort with a genetic algorithm on the “regulation of RhoA activity pathway” gene set (see Methods). The resulting optimal solution, containing 20 genes, is hereafter denoted as RRAP (Figure 1B). These gene mutations were significantly associated with gastric cancer (Supplementary Table 1). According to whether or not mutations occurred in the coding region of any of the RRAP genes, patients were classified as RRAP-wild type or RRAP-mutant. In the TCGA training cohort, RRAP-mutant patients displayed better OS compared to RRAP-wild type patients, with a hazard ratio of 0.4 (95% confidence interval: 0.2-0.79, p-value = 0.006, Figure 2A). Importantly, this was also validated in the TCGA validation cohort, with a hazard ratio of 0.48 (95% confidence interval: 0.26-0.91, p-value = 0.021, Figure 2B). Furthermore, we incorporated age, gender, Lauren classification, pathologic stage, and RRAP into a multivariate Cox analysis for overall survival. The results showed that RRAP was an independent prognostic factor after adjusting for these clinicopathological parameters in both the TCGA training (hazard ratio = 0.35, 95% confidence interval: 0.17-0.7, p-value = 0.003; Supplementary Figure 1A) and TCGA validation cohorts (hazard ratio = 0.51, 95% confidence interval: 0.26-1, p-value = 0.05; Supplementary Figure 1B).

The reliability of RRAP was assessed using an independent patient cohort that was a subset of the 109 CGC patients. These patients were whole-exome sequenced, and 26 (23.9%) were identified as RRAP-mutant. This cohort exhibited a mutational proportion similar to the TCGA training (26.3%) and TCGA validation (28.3%) cohorts. For the reliability assessment cohort, Kaplan-Meier curves showed that RRAP-mutant was significantly associated with longer OS (hazard ratio = 0.13, 95% confidence interval: 0.03- 0.52, p-value = 6.66e-4, Figure 2C). Multivariate Cox regression analysis was performed as described above, and the results again showed that RRAP was an independent prognostic factor (Supplementary Figure 1C). We also merged data of the TCGA training, TCGA validation, and CGC cohorts into overall cohort. RRAP-mutant patients displayed better OS compared to RRAP-wild type patients, with a hazard ratio of 0.38 (95% confidence interval: 0.25-0.59, p-value = 7.37e-6) in the overall cohort (Figure 2D and Supplementary Figure 1D).

### RRAP was associated with cell migration activity

**Figure 3:**
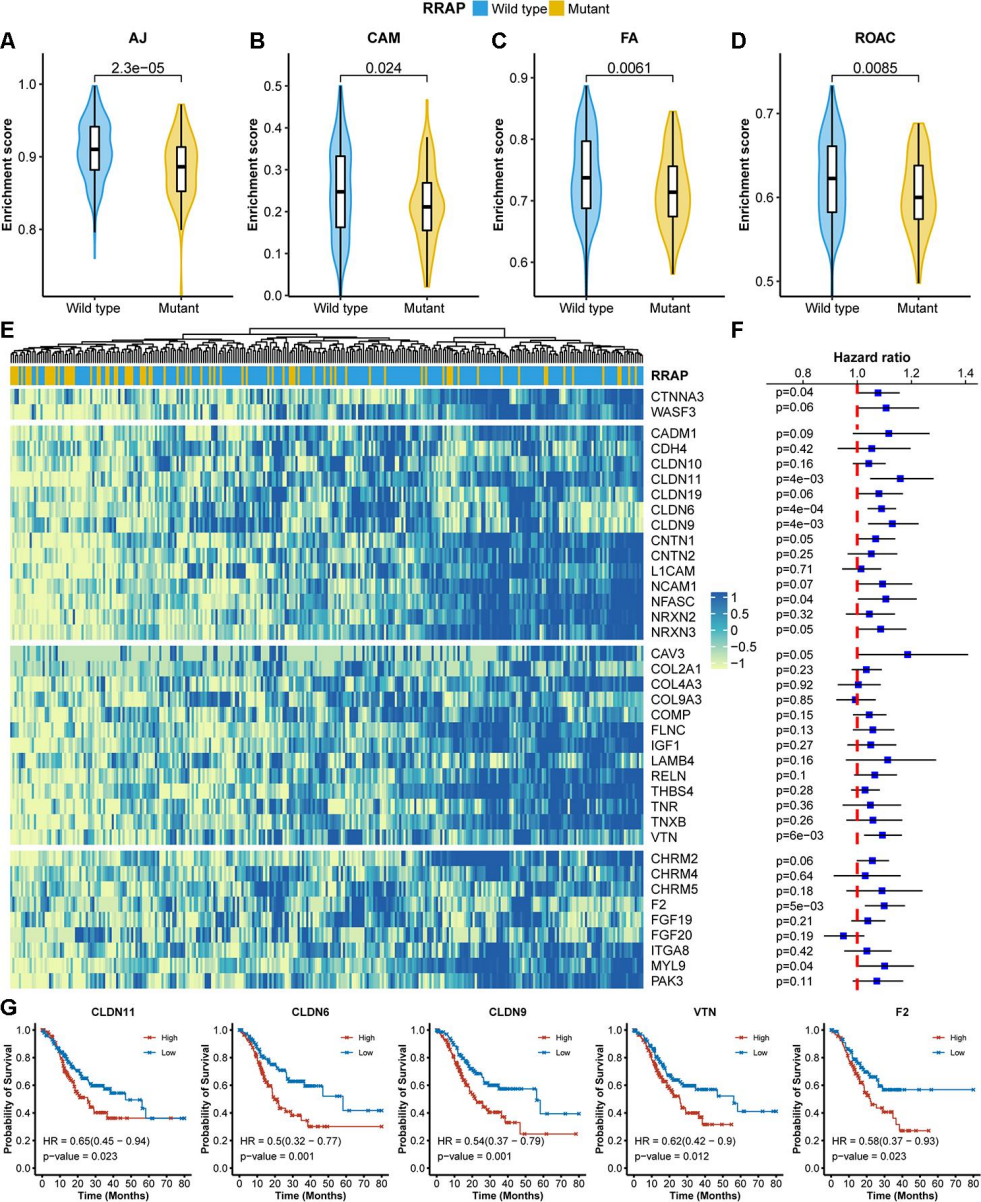
**Association of RRAP with lower cell migration activity.** (**A**–**D**) Pathway enrichment scores between RRAP-wild type and RRAP-mutant were compared among 4 functions: adherens junction (AJ, **A**), cell adhesion molecules (CAM, **B**), focal adhesion (FA, **C**), and regulation of the actin cytoskeleton (ROAC, **D**). The Wilcoxon rank-sum test was applied to estimate differences. (**E**) Heatmap of differentially expressed genes among the 4 functions; the column was clustered and annotated with RRAP status. (**F**) Forest plot of association of between gene expression and overall survival. Squares indicate the hazard ratios and error bars represent the 95% confidence interval; the log-rank test was performed to estimate p-values. (**G**) Kaplan-Meier overall survival curves for patients with high and low expression levels of CLDN11, CLDN6, CLDN9, VTN, and F2. P-values were estimated with the log-rank test, and HRs were estimated with the Cox model.

Given the role of RhoA in cell migration, we assessed the effect of RRAP on tumor metastasis. Strikingly, patients with RRAP-mutant tumors had a significantly lower rate of lymph node metastasis (pN3 of regional lymph node) compared with RRAP-wild type patients (11.5% vs 36.1%, respectively, Fisher's exact test p-value = 0.026) in the CGC cohort, which was also confirmed in the overall TCGA cohort (12.8% vs 24.7%, respectively, p-value = 0.03). We also found that patients with RRAP-mutant tumors had a moderately lower distant metastasis recurrence risk compared to patients with RRAP-wild type tumors in the CGC cohort (15.4% vs 31.3%, respectively, p-value = 0.136); the same trend was observed in the overall TCGA cohort (3.6% vs. 7.9%, respectively, p-value = 0.26). This suggested that RRAP-mutant tumors may have an impaired migration capacity. At the molecular level, we explored the RNA-seq data collected from TCGA and estimated the enrichment score of 4 migration-related functions—adherens junctions, cell adhesion molecules, focal adhesion, and regulation of the actin cytoskeleton. All of these functions showed significantly lower activity in RRAP-mutant tumors compared to RRAP-wild type tumors (Figure 3A–3D). Further analysis revealed that 39 genes related to the 4 functions exhibited significantly lower expression in RRAP-mutant tumors (log2 fold change > 1 and adjusted p-value < 0.01, Figure 3E). We next performed univariate Cox and Kaplan-Meier analysis on the 39 genes and found that 5 genes (*CLDN11*, *CLDN6*, *CLDN9*, *VTN*, and *F2;* Supplementary Table 2) were significantly associated with poor OS (Figure 3F, log-rank p-value < 0.01). Kaplan-Meier analysis revealed that the low expression of these 5 genes, along with *CNTN1*, *CNTN2*, *CADM1*, *NCAM1*, *FGF19*, and *FGF20*, were significantly associated with better OS (Figure 3G, Supplementary Figure 2). Taken together, these results indicated an association between RRAP mutation and tumor cell migration, thus resulting in metastasis.

### Association of RRAP with immune-related signatures

**Figure 4:**
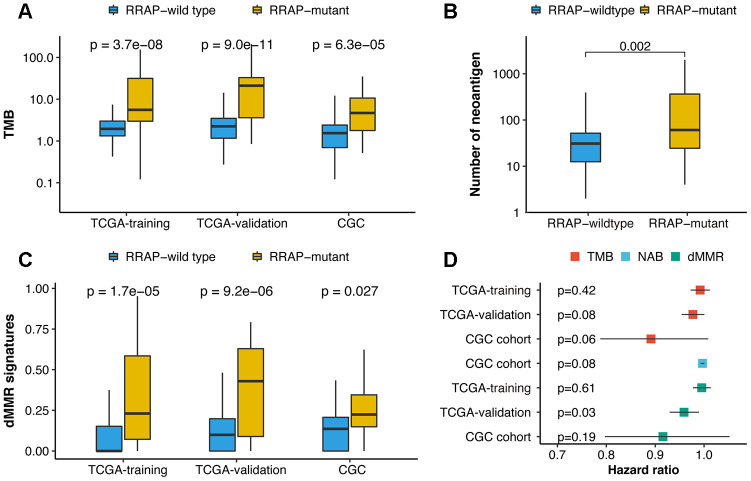
**Association of RRAP with immune-related biomarkers.** (**A**) Boxplot for differences in the tumor mutational burden (TMB) between RRAP-wild type and RRAP-mutant tumors in the TCGA training, TCGA validation, and CGC cohorts. (**B**) Boxplot for differences in the neoantigens between RRAP-wild type and RRAP-mutant tumors in the CGC cohort. (**C**) Boxplot for differences in the dMMR percentage between RRAP-wild type and RRAP-mutant tumors in TCGA training, TCGA validation, and CGC cohort. The Wilcox rank-sum test was applied to compare the differences. (**D**) Association of overall survival and TMB, neoantigen burden (NAB), and dMMR in the 3 cohorts. The hazard ratio was estimated with univariate Cox analysis, and the log-rank test was applied to calculate the p-value.

We next explored the correlation between RRAP and the immune-related signatures of tumor mutational burden (TMB), neoantigen burden (NAB), and deficient mismatch repair (dMMR). Higher TMB was observed in RRAP-mutant patients in the TCGA training, TCGA validation, and CGC cohorts, with p-values of 3.7e-8, 9e-11, and 6.3e-5, respectively (Figure 4A). A significantly higher NAB (available only in the CGC cohort) was observed in RRAP-mutant patients (p-value = 0.002, Figure 4B). Additionally, there was a significantly higher fraction of mutational signatures associated with dMMR in the RRAP-mutant tumors compared to RRAP-wild type tumors in all 3 cohorts (Figure 4C). Although the 3 immune-related signatures in this study could predict the response to immunotherapy in multiple cancers [22], TMB and NAB were not associated with the overall survival of gastric cancer patients, while dMMR was related to overall survival in the TCGA validation cohort (Figure 4D).

### Effect of RRAP mutation on the tumor microenvironment

**Figure 5:**
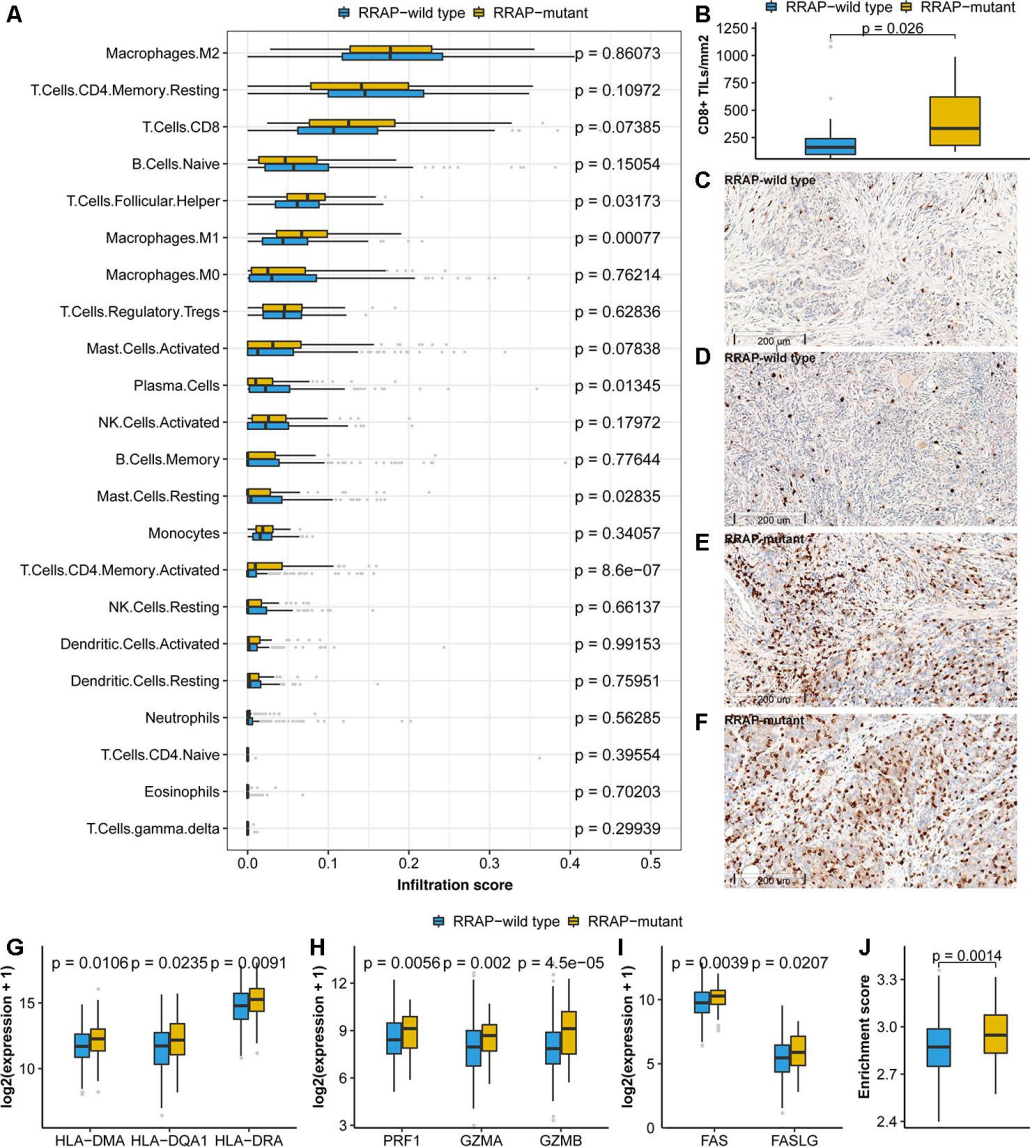
**Association of RRAP with the tumor microenvironment.** (**A**) Immune infiltrations estimated by CIBERSORT were compared between RRAP-wild type tumors and RRAP-mutant tumors. (**B**) The density of CD8+ TILs was compared between RRAP-wild type and RRAP-mutant tumors. (**C**–**F**) The representative immunohistochemistry for CD8 images of RRAP-wild type tumor tissue (**C**, **D**) and RRAP-mutant tumor tissue (**E**, **F**). (**G**–**I**) Expression values were compared for HLA class II genes (**G**), cytotoxic effector molecules (**H**), and apoptosis-related genes (**I**). (**J**) The boxplot shows the difference in enrichment score of the apoptosis pathway between RRAP-wild type and RRAP-mutant tumors. The Wilcoxon rank-sum test was applied to estimate the p-values.

In addition to the 3 signatures above, we further explored the association between RRAP and the TME using TCGA expression data. For this purpose, we collected tumor immune infiltrate data published by TCGA and compared immune cellular fraction between RRAP-mutant and RRAP-wild type tumors [23]. RRAP-mutant tumors showed significantly higher levels of infiltration of activated CD4^+^ memory T cells (p-value = 8.6e-7) and M1 macrophages (p-value = 0.00077) (Figure 5A), both of which are reportedly associated with longer survival in multiple cancers [24, 25]. In addition, we observed higher infiltration of CD8+ T cells—the primary effector in anti-tumor immunity—in RRAP-mutant tumors compared to RRAP-wild type tumors, although it did not reach a statistical significance (p-value = 0.07385, Figure 5A). There was a significantly difference in the levels of CD8+ infiltration in the independent CGC cohort. IHC staining was used to evaluate the infiltration of CD8+ tumor infiltrating lymphocytes (TILs) in 52 tumors from the CGC cohort; 40 of these were RRAP-wild type tumors and 12 were RRAP-mutant tumors. Consistent with the TCGA RNAseq results, RRAP-mutant tumors exhibited increased infiltration of CD8+ TILs compared to RRAP-wild type tumors (p-value = 0.026) (Figure 5B–5F). Given the functional dependence of CD4+ T cells and M1 macrophages on human leukocyte antigen (HLA) class II molecules, further analyses of several HLA class II molecules were also performed. The results showed significantly higher expression of these molecules in RRAP-mutant tumors compared with RRAP-wild type tumors (Figure 5G), including HLA-DMA (p-value = 0.0106), HLA-DQA1 (p-value = 0.0235), and HLA-DRA (p-value = 0.0091). Taken together, an increased anti-tumor immune response by CD4+ T cells and macrophages could be seen for RRAP-mutant tumors.

Granule exocytosis-related molecules (e.g., PRF1/GZMA/GZMB) and death ligand pathways (e.g., the Fas/FasL apoptotic killing pathway) are involved in the cytotoxic effects of CD8+ T cells [26] and possibly of CD4+ T cells [27, 28]. We found that PRF1, GZMA, and GZMB were significantly increased in RRAP-mutant tumors compared to RRAP-wild type tumors, with p-values of 0.0056, 0.002, and 4.5e-5, respectively (Figure 5H). Additionally, RRAP was associated with significantly increased FAS (p-value = 0.0039) and FASLG expression (p-value = 0.0207) (Figure 5I). The apoptosis pathway was also evaluated and exhibited higher activity in RRAP-mutant tumors than in RRAP-wild type tumors (p = 0.0014, Figure 5J). These results strongly suggested that RRAP mutation affected the TME, providing evidence for the association of RRAP-mutant with better OS on the basis of increasing anti-tumor activity in the TME (Supplementary Figure 3).

### RRAP as a potential predictive biomarker for checkpoint inhibitor-based immunotherapy

**Figure 6:**
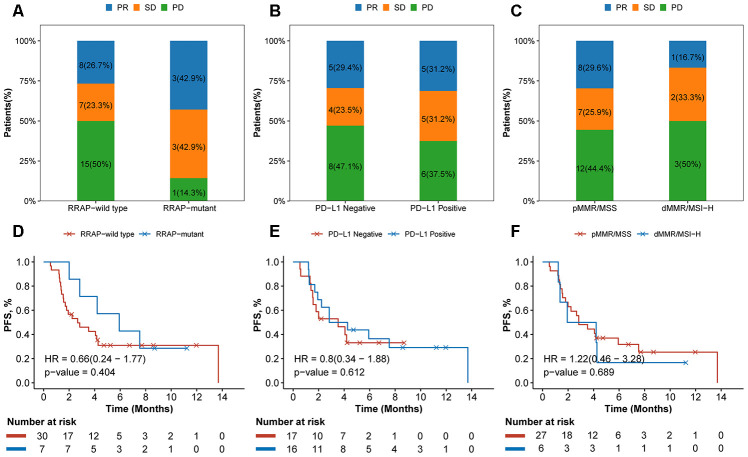
**Association of RRAP and immunotherapy efficacy.** (**A**–**C**) Stacked barplot showing the proportion of patients with progressive disease (PD), stable disease (SD), or partial response (PR) for each group divided by RRAP (**A**), PD-L1 (**B**) and MSI/MMR status (**C**). (**D**–**F**) The text indicates the number and percentage of patients in each group. Kaplan-Meier survival curves for patients grouped by RRAP (**D**), PD-L1 (**E**), and MSI/MMR status (**F**). P-values were estimated with the log-rank test, and HRs were estimated with the Cox model.

Inspired by the significant effect of RRAP on prognosis, immune-related signatures and the TME, we further investigated the association between RRAP and the response to ICB therapy. Of the IM1 cohort who received ICB therapy (N = 37), 7 patients were identified as RRAP-mutant and 30 were RRAP-wild type. After treatment, 15 (50%) patients in the RRAP-wild type group had progressive disease. In contrast, only 1 (14.3%) patient in the RRAP-mutant group had progressive disease (Figure 6A). We also evaluated the efficacy of ICB therapy between groups stratified by PD-L1 expression and microsatellite instability (MSI) and/or mismatch repair (MMR) status, and no notable differences were found (Figure 6B, 6C). The Kaplan-Meier curves suggested that RRAP-mutant favored PFS compared with RRAP-wild type (Figure 6D), with respective median PFS times of 5.93 months (95% confidence interval: 2.83-not available) and 2.67 months (95% confidence interval: 1.70-not available). However, there was no difference in PFS between PD-L1-positive and PD-L1-negative patients (median PFS: 3.55, 95% confidence interval: 1.93-not available and 3.53, 95% confidence interval: 1.57-not available, respectively) (Figure 6E). The same observation was made when comparing dMMR/MSI-H and pMMR/MSS patients (median PFS: 3.07 95% confidence interval: 1.37-not available and 2.83, 95% confidence interval: 2-not available, respectively) (Figure 6F). In the IM2 cohort (N = 47), 12 patients were classified as RRAP-mutant; these patients had a better overall response rate than RRAP-wild type patients (33.3% vs 8.6%, respectively, Fisher's exact test p-value = 0.06). These results suggested that RRAP may play a role as a potential predictive biomarker for checkpoint inhibitor-based immunotherapy in gastric cancer.

## DISCUSSION

In this study, we identified RRAP as a biomarker, validated its prognostic effect in TCGA and CGC gastric cancer data sets, and investigated its association with tumor metastasis, the TME, and its potential prediction value for ICB therapies.

For the TCGA training, TCGA validation, and CGC cohorts, the frequencies of RRAP-mutant were elevated (26.3%, 28.3%, and 23.9%, respectively) compared to *RHOA* mutations alone (7.5%, 4.5%, and 3.7%, respectively), suggesting that RRAP can be more broadly utilized as a predictive biomarker for gastric cancer. RRAP-mutant proved to be an independent prognostic factor and was significantly associated with favorable overall survival, regardless of histological classification (i.e., not only in diffuse gastric cancer). This clinical significance is likely attributed to altered RhoA activity disrupting the process of tumor invasion and metastasis when the process is impaired by RRAP mutation. Clinically, our results showed that patients with RRAP-mutant tumors had a significantly lower rate of lymph node metastasis (pN3) and a lower risk of distant metastasis recurrence. At the molecular level, our analysis suggested that RRAP-mutant tumors exhibited a low activity of migration-related functions (adhesion junctions, cell adhesion molecules, focal adhesion, and regulation of the actin cytoskeleton). In these functions, 11 genes (*CLDN11*, *CLDN6*, *CLDN9*, *VTN*, *F2*, *CNTN1*, *CNTN2*, *CADM1*, *NCAM1*, *FGF19*, and *FGF20*) that were downregulated in RRAP-mutant tumors were significantly associated with improved OS. Our findings are supported by previous results, not only regarding the role of RhoA in tumor cell invasion and metastasis [29] but also regarding the association of RhoA activity with gastric cancer prognosis [30, 31].

Recent efforts have shown that RhoA can modulate the TME [8, 9]. Therefore, we further assessed the significant association between RRAP and clinical outcomes in terms of the TME. Our results indicated that RRAP-mutant patients had higher fractions of activated CD4+ memory T cells, CD8+ T cells, and M1 macrophages, all of which have been reported as biomarkers that are positively associated with overall survival in multiple cancers [24, 25]. We further found that HLA-DMA, HLA-DQA1, and HLA-DRA, on which CD4+ T cells and M1 macrophages functionally depend [32, 33], also showed significantly increased expression in RRAP-mutant tumors. Our experimental CD8+ IHC results were highly consistent with our bioinformatics analysis. Moreover, we also showed that mutated RRAP may affect the TME by regulating the expression levels of granule exocytosis-related molecules (PRF1/GZMA/GZMB) and the death ligand pathway (Fas/FasL apoptotic killing pathway), which are involved in the cytotoxic effects of CD8+ T cells and possibly of CD4+ T cells. Taken together, these results strongly indicated an association between RRAP-mutant and better OS on the basis of increasing antitumor activity in the TME (Supplementary Figure 3). The association between well-known immunotherapy-related biomarkers (TMB, NAB, and dMMR) and the RRAP status was also assessed. Interestingly, all three biomarkers were significantly higher in RRAP-mutant tumors; however, only RRAP had prognostic significance. Moreover, RhoA signaling plays an important role in inducing activating innate immune and adaptive T cell responses [34]. For example, the downregulation of CDC42 reduces NK cell-mediated killing, allowing cancer cells to escape from the human immune response [35]. VAV3, a Rho family GTPase, activates multiple cell signaling pathways, including NK cell activation [36]. And the role of RhoA for phagocytosis has been studied in macrophages [37, 38]. Besides, RhoA signaling in T cells and B cells is pivotal for activation and migration [34]. The association of RRAP-mutant with immune activation and anti-tumor activity in the tumor immune microenvironment may provide clues to the predictive effect of RRAP in immunotherapy response.

The US Food and Drug Administration approved MSI-H/dMMR as a biomarker for immunotherapy; MSI-H/dMMR occurs in only 4-22% of gastric cancer cases [3, 39, 40] with an approximately 40-57% response [40, 41]. Many studies have shown that TMB, CD8+ TILs, and PD-L1 expression correlate with the efficacy of immunotherapy [42–44]; however, the association between these markers and the clinical benefit of gastric cancer immunotherapy is uncertain [45]. Accordingly, in the current study, 2 retrospective analyses on the IM1 and IM2 cohorts were performed to predict the efficacy of ICB therapy based on RRAP status. The response rate of RRAP-mutant patients was higher than that of RRAP-wild type patients within both the IM1 and IM2 cohorts. The differences are expected to be significant in a larger sized cohort. A clinical trial based on the RRAP biomarker is being designed. Our findings indicate that RRAP plays an important role in the regulation of the TME. Its status as a predictive biomarker will be further verified in larger clinical cohorts receiving immunotherapy.

In summary, this is one of the few efforts of biomarker identification for disease prognosis and therapeutic response based on pathway genomic characteristics in gastric cancer. In gastric cancer patients, RRAP-mutant tumors were correlated with a better prognosis, regardless of the histological classification and clinicopathological parameters. We investigated this correlation mainly from the perspective of RRAP regulating tumor invasion, metastasis, and the TME. Patients with RRAP-mutant tumors showed a better response to checkpoint inhibitor-based immunotherapy. These findings shed light on the clinical implications of the RRAP-mutant biomarker and may be used to guide personalized therapy for gastric cancer patients.

## MATERIALS AND METHODS

### Gastric cancer cohorts

The 4 gastric cancer patient cohorts included in this study were the TCGA cohort (N=315, Supplementary Table 3), the CGC cohort (N = 109, Supplementary Table 3), and 2 immunotherapy cohorts, IM1 (N = 37) and IM2 (N = 47). Clinical and mutation data from the TCGA database (N = 440) were downloaded from the cBioportal database [46], and survival data were collected from a previous study [47]. Only patients with American Joint Committee on Cancer (AJCC) version 8 stage II and III were included (N = 319). Four patients without a survival time were excluded; the final number of patients in the TCGA cohort was 315. The TCGA training (N = 160) and validation (N = 155) cohorts were generated by random sampling to be approximately equal in sample size. Formalin-fixed, paraffin-embedded tumor tissue samples from the CGC cohort were collected from 109 stage II–III treatment-naive gastric cancer patients who had primary gastric cancer resection at Peking University Cancer Hospital and Institute between 2008 and 2015. The tumor tissue samples of the IM1 cohort were obtained from metastatic gastric cancer patients before they started ICB treatment. After the samples were collected, these 37 patients received at least 1 cycle of any ICB therapy regardless of the agent’s target (i.e., PD-1/PD-L1). The IM2 cohort containing 47 patients who received PD-1 inhibitor therapy (toripalimab) was collected from multiple centers, details of which were previously published [48].

This study was approved by the medical ethics committee of the Peking University Cancer Hospital, and participants provided informed consent. The design and implementation of the study complied with the local regulations and guidelines and with the basic principles of the Declaration of Helsinki.

### Whole-exome sequencing analysis and variant filtering

Tumor tissue and adjacent nontumor tissue samples from CGC cohort patients were subjected to whole-exome sequencing. All whole-genome sequencing (including DNA extraction and quality control) was performed in the OrigiMed laboratory. This laboratory is College of American Pathologists-accredited and is a Clinical Laboratory Improvement Amendments (CLIA) certified laboratory. All tumor tissue slides were reviewed by two independent pathologists, and samples with estimated tumor purity greater than 20% were included in the study. In detail, DNA was extracted from the formalin-fixed, paraffin-embedded samples according to the manufacturer's instructions. Next, ~ 500 ng of genomic DNA was sheared to a mean fragment length of 200 bp and labeled with a 6-8 base barcode during polymerase chain reaction (PCR) amplification. Exomes were captured using a SureSelectXT Human All Exon V6 (Agilent Technologies). Sequencing was performed with an Illumina HiseqX instrument using 150 base paired-end reads. Reads were trimmed with AdapterRemoval v2 [49], aligned to the human reference genome (GRCh37) by Burrows-Wheeler Aligner v0.7.5a [50], and PCR duplicates were removed by Picard (version 1.47). Somatic variants were identified by GATK4 Mutect2 (version 4.0.6) [51] and then annotated with SnpEff (version 4.3b) [52]. Variants in the common dbSNP database (version 147) or those having a frequency above 1.5% in the Exome Sequencing Project 6500 or 1000 genome project were excluded from further consideration. The variant files have been uploaded to the European Variation Archive (PRJEB31906). Low-confidence variants in both the TCGA and CGC cohorts were removed by applying the following filters: (1) total coverage < 30, (2) variant allele depth < 7, and (3) variant allele frequency < 0.05.

### Feature selection and biomarker validation

The prognostic biomarker was calculated by a genetic algorithm that was implemented in the Python package pyeasyga [50, 53]. In brief, each solution was a binary vector with a 0 or 1 value that had the same length as the original full set of the “regulation of RhoA activity pathway” biomarker (length = 48); 1 indicated that the corresponding gene harbored a nonsynonymous substitution or indel in the coding region, and 0 indicated neither of these changes. The goal was to find the best solution containing the genes with their values equal to 1, which was considered as the optimal gene subset (i.e., biomarker). The parameters of the genetic algorithm were empirically set as follows: a total population size of 40 solutions, crossover probability of 0.1, mutation probability of 0.01, and max generation of 50 000. The fitness value was calculated as follows: a cohort that was randomly selected (size = 80, 50%) from the 160 patients of the TCGA training cohort was further divided into a mutation and wild-type group according to the mutation status of the genes that corresponded to value 1 of each solution; a log-rank p-value was calculated for this division, the whole process was performed 100 times, and the mean p-values were calculated as the fitness value. Finally, the best solution was generated, and the resulting genes with a value of 1 were regarded as the final subset (i.e., the biomarker).

### Tumor mutational burden, neoantigens, and mutational signatures

The tumor mutational burden (TMB) was estimated by dividing the total number of somatic variants by the coding region size. All somatic variants in the coding and splicing regions were counted, and the coding region size was estimated as 33 Mb based on RefSeq Genome Annotation (GRCH37). HLA typing was performed with OptiType (version 1.2.1) [54]. All nonsynonymous mutations that were identified were translated into peptides of 17 amino acids with an in-house pipeline. The sliding window (approximately 9-11 amino acids in size) method was used to identify substrings within the 17mer that had a predicted HLA class I binding affinity of less than 500 nM to any patient-specific HLA allele. The binding affinity for amino acids and alleles was analyzed using NetMHCpan v3.0 [55]. The neoantigen burden was estimated as the total number of substrings with a binding affinity less than 500 nM. Mutational signature contributions were identified using the R package deconstrucSigs (version 1.8.0) [56] with 30 signatures documented by the Catalogue of Somatic Mutations in Cancer as a reference [57]; samples with an error rate less than 0.15 and mutation counts greater than 30 were considered for mutational signature analysis. Deficient mismatch repair was estimated as the sum of signature 6, 15, 20, and 26 [57].

### RNA-seq data analysis

RNA-seq data of the TCGA cohort were extracted with TCGAbiolinks [58]. Raw read counts were normalized with DEseq2 [59] and then log2 transformed. The ssGSEA algorithm in the R package GSVA [60] was used to evaluate the pathway enrichment score. Kyoto Encyclopedia of Genes and Genomes (KEGG) pathway gene sets were retrieved from the KEGG database by the R package gage [61].

### Immunohistochemistry staining and evaluation

Immunohistochemistry (IHC) analysis for CD8 (clone SP16, ZSGB-BIO) was evaluated within intratumor areas. Aperio Scanscope (Aperio Technologies Vista, CA, USA) was used to quantify CD8+ density by the rare event tissue test method. We counted the total number of CD8+ cells of each area based on 6 randomly captured visual fields (400×400 m^2^) and defined the density of CD8+ TILs as the total cell number per square millimeter. IHC staining of anti-PD-L1 (clone SP142, Spring Bioscience) was also annotated within intratumor areas. The percentages of cancer cells and immune cells with anti-PD-L1 staining were measured in each area based on 3 visual fields in darkly stained areas (400×400 m^2^). The expression of PD-L1 was defined as positive when ≥ 1% of the tumor/stromal cells were positive. IHC-stained sections were scored independently by 2 gastrointestinal pathologists blinded to the clinicopathological parameters and biomarker results.

### Mismatch repair/microsatellite instability testing

To evaluation the mismatch repair (MMR) and/or microsatellite instability (MSI) status, MLH1 (clone ES05, Gene Tech), MSH2 (clone 25D12, Gene Tech), MSH6 (clone EP49, Gene Tech) and PMS2 (clone EP51, Gene Tech) were stained. The complete loss of expression of one or more proteins was considered as dMMR. In some cases, the microsatellite stability status was calculated by using a single multiplex PCR that assessed five microsatellite loci (BAT-25, BAT-26, D2S123, D5S346, and D17S250) [62]. For interpretation, instability at more than one locus referred to high microsatellite instability (MSI-H), instability at a single locus referred to low microsatellite instability (MSI-L), and no instability at any locus referred to stable microsatellites (MSS) [63].

### Statistical analysis

Survival analysis was carried out by the Kaplan-Meier method, and the difference between the groups was compared using the log-rank test. The hazard ratio and the 95% confidence interval were estimated by the Cox model. The multivariate Cox model was performed to adjust for confounding factors. Fisher’s exact test was used to compare proportions. The Wilcoxon rank-sum test was used to compare continuous values. All statistical analyses were performed with R software 3.5.3.

## Supplementary Material

Supplementary Figures

Supplementary Table 1

Supplementary Tables 2 and 3
