# Supplementary material for: Identification of “regulation of RhoA activity panel” as a prognostic and predictive biomarker for gastric cancer
Source: Aging (Albany NY). 2020 Dec 3;13(1):714–34. doi: 10.18632/aging.202179 (PMC7835016; doi:10.18632/aging.202179)
Supplement: Supplementary Figures [file aging-13-202179-s001.pdf]

SUPPLEMENTARY FIGURES

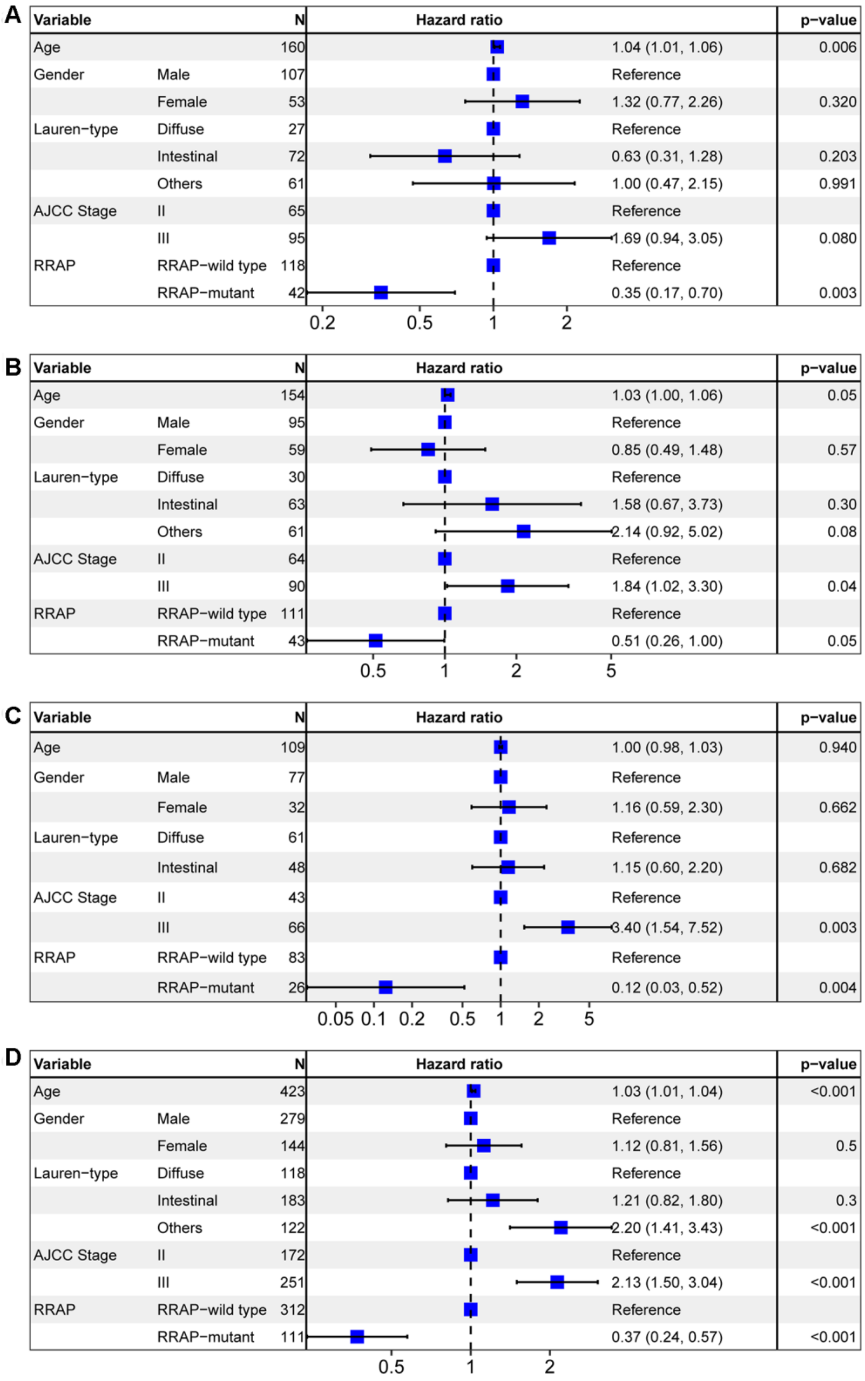

Supplementary Figure 1. Multivariate Cox model analysis, with age, gender, Lauren type, AJCC stage, and RRAP as confounding factors, performed on the TCGA training cohort (**A**), TCGA validation cohort (**B**), CGC cohort (**C**), and the overall cohort containing merged data from the three cohorts (**D**).

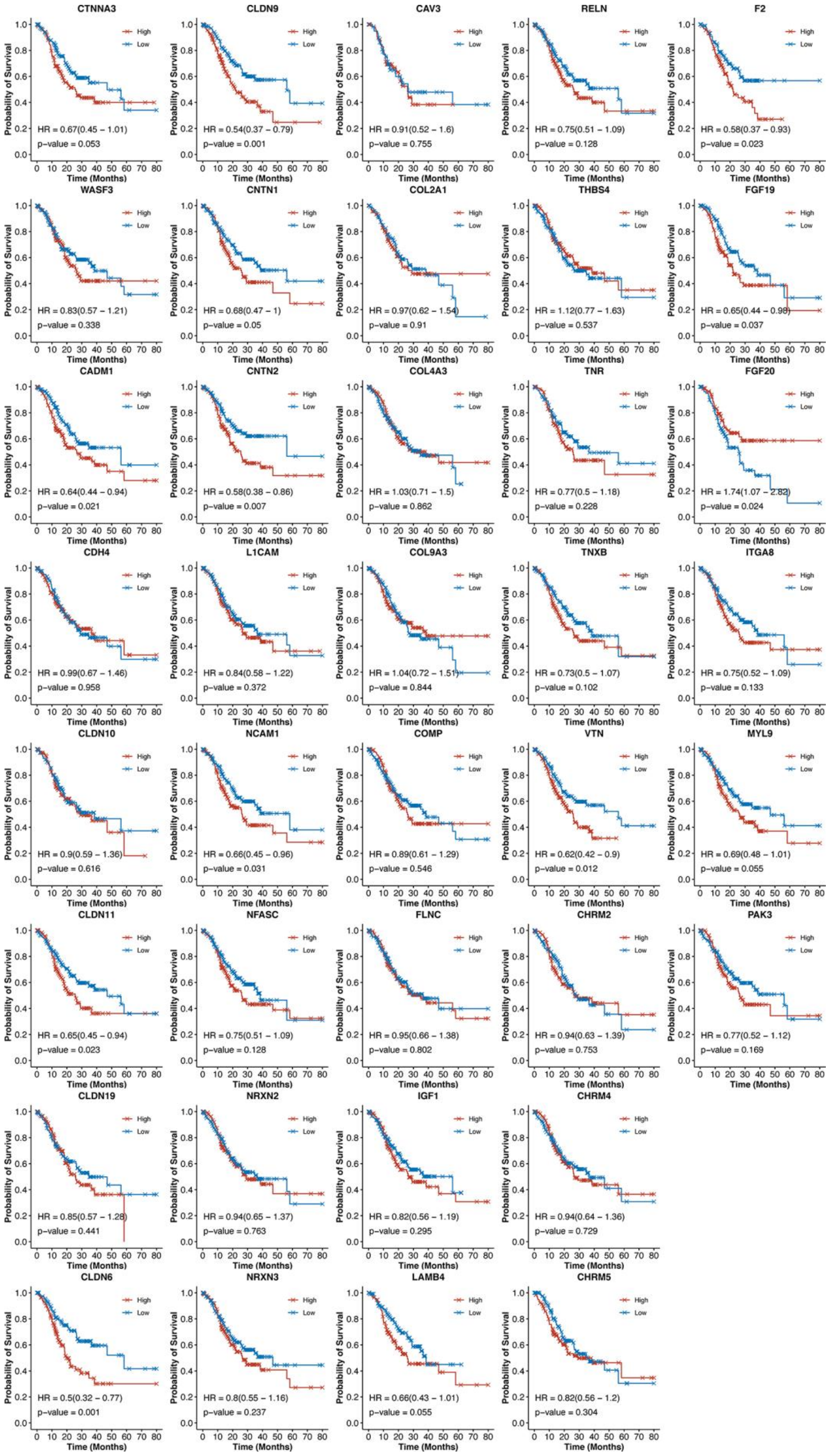

Supplementary Figure 2. Kaplan-Meier overall survival curves for patients with high and low expression of 39 differentially expressed genes of the 4 migration-related functions. P-values were estimated with the log-rank test, and hazard ratios were estimated with the Cox model.

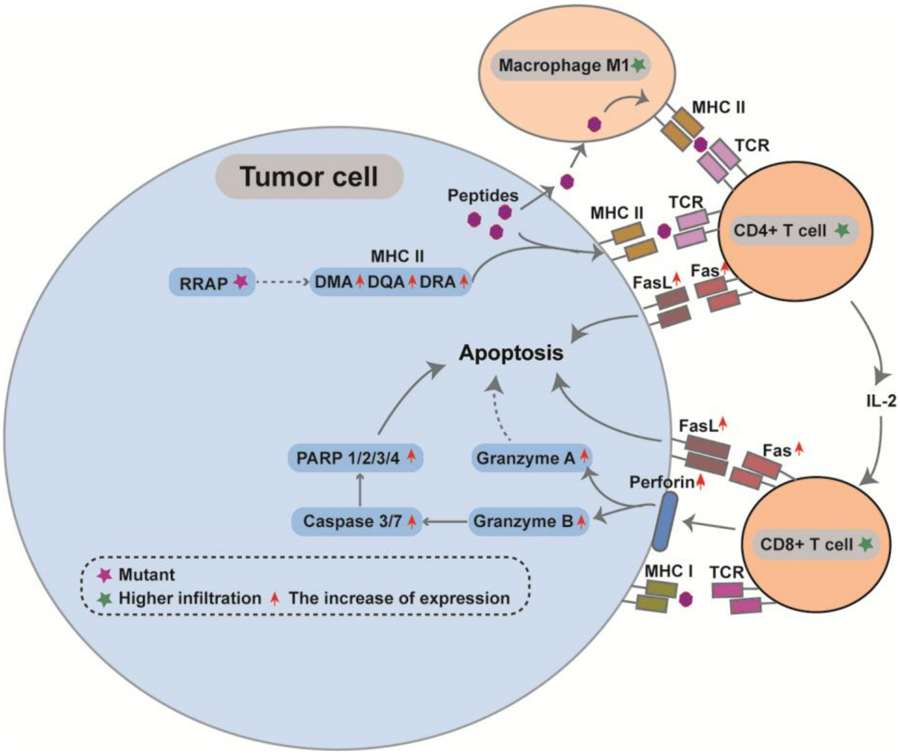

Supplementary Figure 3. Diagram of the interaction of immune cells and tumor cells in the RRAP-mutant tumor microenvironment.
