# Supplementary material for: Identification of “regulation of RhoA activity panel” as a prognostic and predictive biomarker for gastric cancer
Source: Aging (Albany NY). 2020 Dec 3;13(1):714–34. doi: 10.18632/aging.202179 (PMC7835016; doi:10.18632/aging.202179)
Supplement: Supplementary Tables 2 and 3 [file aging-13-202179-s003.pdf]

SUPPLEMENTARY TABLES

Please browse Full Text version to see the data of Supplementary Table 1.

Supplementary Table 1. Genes contained in the RRAP biomarker gene set and their mutation frequency in the TCGA training, TCGA validation, and CGC cohorts, along with migration activity.

Supplementary Table 2. The CLDN11, CLDN6, CLDN9, VTN, and F2 with cell migration activity.

Symbol	Conclusion	Experiment model	PMID
CLDN6	Higher **CLDN6** expression promotes the cell proliferation and migration ability of gastric cancer. Increased expression of **CLDN6** predicts poor prognosis in gastric cancer patients.	MKN28, AGS, MKN7, NUGC-3, xenograft subcutaneous tumor model	31827075, 20874001, 27914788, 31654186
CLDN9	**CLDN9** overexpression in gastric adenocarcinoma cells has been reported to increase cell migration and proliferation. The expression of **CLDN9** in gastric cancer correlates to poor prognosis. Higher **CLDN9** expression promotes invasiveness of several solid tumors in vitro and metastasis in vivo.	AGS, HL7702, a spontaneous metastasis model	20874001, 29031421, 31418417, 26669782
CLDN11	Silencing of **CLDN11** is associated with increased invasiveness, proliferation, and migration of gastric cancer cells. DNA hypermethylation is associated with the downregulation of **CLDN11** in gastric cancer cells.	MKN28, MKN74, AGS, HFE145	32119960, 28962204, 19956721
F2	**F2** is generated in the TME, promoting the migration and metastasis of tumor cells.	B16, HT29-D4, SUM149, MC38 cells	30654498, 15539922, 12707033, 23280128
VTN	Decreased expression of **VTN** promotes gastric cancer cell growth and metastasis. **VTN** is related to the migration and invasion of other solid tumors, such as ovarian adenocarcinoma, fibrosarcoma and nasopharyngeal carcinoma.	AGS, IGROV1, HT-1080,5-8F, HNE2, HONE1 cells	25789040, 9935212, 16052409, 29123267

Supplementary Table 3. Clinical characteristics in TCGA-training, TCGA-validation, and CGC cohorts.[a]

Characteristic	TCGA- training (N=160)	TCGA-validation (N=155)	CGC (N=109)
Age, years			
Mean (SD)	64.3 (10.8)	66.2 (9.9)	57.9 (13.0)
Median (Q1 – Q3)	66 (57-72)	68 (58-73)	58 (51-67)
Min – Max	30-84	43-90	25-83
Gender - no. (%)			
Male	107 (66.9%)	96 (61.9%)	77 (70.6%)
Female	53 (33.1%)	59 (38.1%)	32 (29.4%)
AJCC Stage - no. (%)			
II	65 (40.6%)	65 (41.9%)	43 (39.4%)
III	95 (59.4%)	90 (58.1%)	66 (60.6%)
Lauren classification - no. (%)			
Diffuse	27 (16.9%)	30 (19.4%)	61 (56.0%)
Intestinal	72 (45.0%)	64 (41.3%)	48 (44.0%)
Unknown	61 (38.1%)	61 (39.3%)	—
Tumor location - no. (%)			
Gastroesophageal junction	33 (21.0%)	31 (20.0%)	34 (31.2%)
Non-gastroesophageal junction	123 (76.9%)	121 (78.1%)	75 (68.8%)
Unknown	4 (3.0%)	3 (2.0%)	—
Adjuvant chemotherapy - no. (%)			
Yes	81 (50.6%)	70 (45.2%)	98 (90.0%)
No	—	—	8 (7.3%)
Unknown	79 (49.4%)	85 (54.8%)	3 (2.7%)

[a]Because of rounding, percentages may not total 100.
